# How do cultural ecosystem services affect visitor emotions? Evidence from social media data in Shanghai urban parks

**DOI:** 10.3389/fpsyg.2025.1711498

**Published:** 2026-01-09

**Authors:** Shuhan Zhou, Yuxiang Liu

**Affiliations:** 1 Edinburgh School of Architecture and Landscape Architecture, University of Edinburgh, Edinburgh, United Kingdom; 2 School of Architecture and Landscape Architecture, University of British Columbia, Vancouver, BC, Canada

**Keywords:** cultural ecosystem services, LDA topic modeling, sentiment analysis, social media reviews, urban parks

## Abstract

**Introduction:**

Traditional Cultural Ecosystem Services (CES) assessments rely largely on questionnaires or interviews, which are limited in capturing large-scale user perceptions and often lack classification frameworks suitable for Chinese linguistic and cultural contexts. With increasing demand for high-quality urban park experiences, understanding how CES shape visitors’ emotional responses has become essential for park planning and management.

**Methods:**

Using social media review data from five representative urban parks in Shanghai, this study developed an integrated analytical framework that combines LDA topic modeling, a CES classification system adapted to Chinese contexts, sentiment analysis (SnowNLP and BERT), and linear mixed-effects modeling (LMM). A total of 22,564 valid user reviews were processed through text cleaning, segmentation, topic extraction, CES semantic classification, and sentiment quantification to evaluate how different CES types influence emotional outcomes.

**Results:**

Five CES categories were identified: Recreation and Family Engagement, Symbolic and Inspirational Landscapes, Physical and Mental Well-being, Aesthetic and Emotional Experience, and Education and Cognitive Engagement. Recreation and Family Engagement and Symbolic and Inspirational Landscapes were the most frequently perceived CES across parks, while Education and Cognitive Engagement appeared least often. Sentiment analysis showed overwhelmingly positive emotional expression, with “Very Positive” exceeding 83% of all reviews and displaying clear seasonal patterns—higher positivity in spring and autumn, and lower scores in summer and winter. LMM results indicated that Recreation and Family Engagement and Symbolic and Inspirational Landscapes significantly enhanced positive emotions, whereas Education and Cognitive Engagement showed a weak negative association in certain contexts.

**Discussion:**

The findings demonstrate the effectiveness of social media text analysis for large-scale CES quantification and highlight the differentiated emotional impacts of various CES types. This integrated LDA–CES–sentiment–LMM framework provides methodological innovation for data-driven CES assessment and offers practical insights for emotion-sensitive urban park planning, visitor experience enhancement, and the design of culturally responsive public spaces.

## 1 Introduction

To accommodate growing populations, urban spaces are often forced to expand outward or densify inward (Desa, 2014). However, in the absence of effective planning and regulation, such processes can trigger uncontrolled construction land sprawl, compressing residents’ living space and exacerbating the mismatch between the supply and demand of public resources. Excessive urban expansion further contributes to declining environmental quality, weakened ecological functions, and worsening traffic congestion, among other adverse outcomes (Liu et al., 2023; Puplampu and Boafo, 2021). As vital components of urban green infrastructure, urban parks not only deliver ecological services—such as moderating urban temperatures and managing stormwater—but also function as key venues for recreation, social engagement, and cultural experience, thereby helping improve environmental conditions and enhance residents’ quality of life (Opdam, 2020; Tost et al., 2019; Wolch et al., 2014). In the post-pandemic era, urban green spaces, particularly parks, have assumed increased importance, accompanied by growing public expectations for diverse functions, amenities, and experiential qualities (Cheng et al., 2021; Spotswood et al., 2021; Stessens et al., 2020). Despite the continuous increase in both the number and total area of urban parks, many visitors still report unsatisfactory on-site experiences—such as monotonous landscapes, inadequate management, and signs of environmental decline (Wan et al., 2024). These challenges frequently surface in social media discourse and news reports, highlighting an urgent need for improved park design and management to mitigate negative emotional responses and foster public well-being (Jang et al., 2024). Consequently, gaining a deeper understanding of users’ experiences and subjective perceptions is essential for the scientific planning, construction, and governance of urban parks that more effectively align with public needs.

The Millennium Ecosystem Assessment (Corvalan et al., 2005) defines Cultural Ecosystem Services (CES) as the non-material benefits people obtain from ecosystems through spiritual enrichment, cognitive development, and other means. These services encompass recreation and ecotourism, aesthetic values, cultural heritage, environmental education, and spiritual or religious benefits. CES are often directly perceived and appreciated by the public and exhibit complex interactions with other categories of ecosystem services, making them especially pertinent to processes of urban development, renewal, and human well-being. In recent years, scholars worldwide have examined CES from social, spatial, and behavioral perspectives, addressing topics such as perception and satisfaction assessments (Wang et al., 2021b; Zhang et al., 2025b), trade-offs and synergies (Adhikary et al., 2025; Zhang et al., 2025a), and value quantification and spatial mapping (Clemente et al., 2019). With the widespread integration of social media into daily life (Fisher et al., 2019; Gosal and Ziv, 2020; Lee et al., 2019; Zhang et al., 2025a), user-generated content—such as comments, photographs, and emotional expressions—has emerged as a valuable new data source for CES research (Cao et al., 2022; Fox et al., 2021; Wang et al., 2025). Analyses of such data provide effective insights into public perceptions, emotional responses, and preferences related to non-material ecosystem benefits (Fernandez et al., 2022; Fox et al., 2021; Huai et al., 2023). Compared with traditional surveys and interviews, social media data offer advantages of large sample coverage, lower cost, timeliness, and convenience, enabling a more dynamic and fine-grained understanding of human–CES interactions. For example, Li et al. (2025) analyzed social media sentiments toward urban parks and demonstrated that visitors’ negative emotional responses were jointly shaped by internal landscape features—such as vegetation quality and facility conditions—and external built-environment factors including accessibility and surrounding urban density. Similarly, Kong et al. (2022) showed through sentiment analysis of social media data that visitors’ positive sentiment varied significantly across park types, with positive correlations to park size and water bodies and negative correlations to impervious surfaces. Despite substantial progress in CES classification, assessment, and valuation, the field continues to face persistent challenges stemming from the intangible, subjective, and context-dependent nature of CES (Adhikary et al., 2025). These challenges include inconsistent evaluation standards and methodological difficulties in quantifying non-material benefits (Dușcu and Rîșnoveanu, 2025; Ebner et al., 2022). Consequently, leveraging emerging data sources and advanced analytical techniques for more accurate identification and quantification of CES remains a pressing scientific priority.

Moreover, social media texts often convey emotions and experiences through vivid, location-specific, first-person narratives. The construction of perception lexicons and the adoption of semantic-mining techniques have become important approaches for examining sustainable urban development and green-space experiences (Wang et al., 2021a; Xu et al., 2025). Advances in data mining and text analytics have enabled researchers to quantify public perceptions and emotions associated with specific landscape features, thereby revealing user preferences and elucidating how these features shape subjective experiences (Liang and Zhang, 2021). Natural language processing techniques—such as topic modeling—are particularly effective for uncovering latent semantic structures and have been extensively applied to multi-source big data, including online reviews and news reports. These methods provide new analytical pathways for understanding public sentiment, information diffusion, and CES typologies. However, research on CES perception and emotional responses derived from text data remains relatively limited. On one hand, mining CES-related information from Chinese social media platforms is still in its early stages, and systematic, high-quality CES lexicons and classification frameworks tailored to colloquial, context-dependent expressions remain scarce. On the other hand, many existing studies rely solely on simple sentiment polarity measures, making it difficult to achieve fine-grained CES identification or to quantitatively assess how different CES types influence public emotions (Huai and Van de Voorde, 2022).

Furthermore, as emotional, aesthetic, and cognitive values emerging from human–nature interactions, CES serve as important mediators for understanding the relationship between nature-based experiences and emotional responses. Environmental psychology provides a foundational theoretical basis for this relationship. Kaplan’s Attention Restoration Theory (ART) (Kaplan, 1995) posits that natural environments promote attentional recovery through restorative qualities such as “soft fascination,” “being away,” and “extent,” thereby facilitating the generation of positive emotions after contact with natural settings. Ulrich et al.’s (1991) Stress Reduction Theory (SRT) further suggests that natural elements can rapidly reduce physiological stress and generate immediate emotional benefits. Correspondingly, many CES categories—such as aesthetic appreciation, symbolic landscapes, and immersive nature experiences—align with the restorative stimuli emphasized in ART and SRT. Meanwhile, emotional geography (Davidson et al., 2012) underscores that emotions are deeply embedded in specific spatial contexts and social interactions. Urban parks function not only as natural backdrops but also as “emotional places,” shaped through activities such as recreation, social interaction, learning, and family engagement. Based on these theoretical foundations, examining “how different CES types influence visitors’ emotions” is not only practically meaningful but also provides an important analytical lens for understanding psychological effects within green spaces.

In recent years, research on CES has expanded substantially, with scholars examining CES from diverse perspectives. For example, studies have analyzed the spatial supply of CES and differences in stakeholder perceptions through the lenses of landscape patterns, ecological valuation, and tourism or recreational environments (Adhikary et al., 2025; Chen et al., 2025; Zhang et al., 2025a). In cultural landscape and heritage contexts, deep learning and social media data have been employed to identify emotional landscapes and explore the affective characteristics and underlying mechanisms of CES (Ren et al., 2025). Nevertheless, research that specifically focuses on CES perception mechanisms within urban parks—everyday, high-frequency public spaces—remains comparatively limited. There is still a lack of systematic understanding of how the public expresses cultural experiences in real social contexts and how different CES types shape emotional responses. Consequently, fine-grained identification of CES in urban parks is insufficient, and the emotional pathways associated with different CES categories remain underexplored. Meanwhile, although several CES lexicons and classification frameworks have been developed within Chinese linguistic and cultural contexts in recent years, most of these tools were designed for structured data scenarios such as questionnaires, landscape annotation, or image analysis. Their applicability to the colloquial, implicit, and context-dependent expressions commonly found in social media text is therefore limited. Moreover, studies employing large-scale user-generated content (UGC) for multi-level statistical testing and temporal variation analysis remain scarce. Overall, existing research has yet to fully reveal the structural characteristics of CES perception or the spatiotemporal dynamics of CES-related emotional effects in urban parks.

To address these gaps, this study examines five representative urban parks in Shanghai and develops an integrated analytical framework that combines LDA topic modeling, keyword frequency statistics, and expert semantic annotation to construct a CES classification scheme tailored to the linguistic and experiential characteristics of Chinese social media text. Rather than replacing existing CES lexicons, this approach extends and contextually adapts them to better capture the implicit, colloquial, and experience-oriented expressions commonly found in UGC. Furthermore, by linking the LDA-identified CES categories with sentiment indicators through linear mixed-effects models (LMMs), this study establishes a systematic and replicable method for quantifying the emotional impacts of different CES types. Taken together, this integrated LDA–LMM workflow addresses several key methodological challenges—such as limited CES recognition in park contexts, the lack of quantitative evidence on emotional mechanisms, and the semantic ambiguity inherent in Chinese UGC—and provides methodological innovation and empirical support for advancing data-driven CES assessment and emotion-sensitive urban park planning.

## 2 Materials and methods

### 2.1 Study area

**Figure 1:**
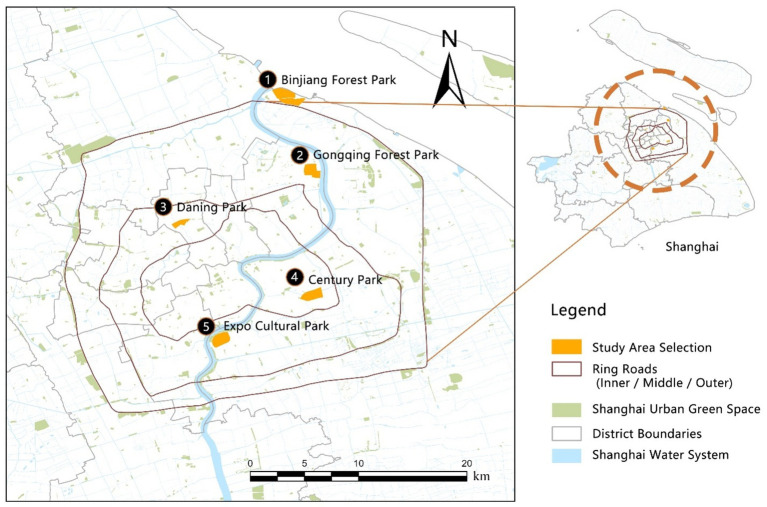
Distribution of study area and sample parks in Shanghai.

Against the backdrop of rapid urbanization, Shanghai has been actively advancing its “park city” initiative, developing a diverse and multifunctional system of urban parks that provide essential spaces for recreation, ecological experiences, and cultural activities. This study focuses on five representative large-scale urban green spaces in Shanghai—Daning Park (54.11 ha), Gongqing Forest Park (122.8 ha), Century Park (131.54 ha), Expo Culture Park (131.95 ha), and Binjiang Forest Park (110.43 ha). The selection of these parks was based on three criteria: (1) Scale and functional diversity: All five parks have substantial spatial extent and heterogeneous landscape structures, collectively representing the major urban park typologies in Shanghai, including comprehensive urban parks (Daning Park and Century Park), forest- and ecology-oriented parks (Gongqing Forest Park and Binjiang Forest Park), and a newly developed large-scale urban park with prominent waterfront characteristics (Expo Culture Park). (2) Spatial representativeness: The parks are distributed within or around Shanghai’s Outer Ring Road, ensuring coverage across multiple urban spatial contexts. Century Park and Expo Culture Park are located within the Outer Ring; Daning Park is situated in the northern part of the central city; while Gongqing Forest Park and Binjiang Forest Park are positioned near the periphery of the Outer Ring. This spatial configuration spans core urban areas to peri-urban zones. (3) Variation in development periods: The selected parks reflect different stages of Shanghai’s urban development, ranging from Gongqing Forest Park, established in the late 20th century (1986), to Expo Culture Park, constructed in the post-Expo era (2021). This temporal diversity captures the evolution of park-planning philosophies, ecological priorities, and landscape design approaches over time. Overall, these five parks are highly representative in terms of scale, ecological structure, and spatial context, providing a robust empirical foundation for examining public perceptions of CESs and their emotional outcomes (see Figure 1).

### 2.2 Technical roadmap

**Figure 2:**
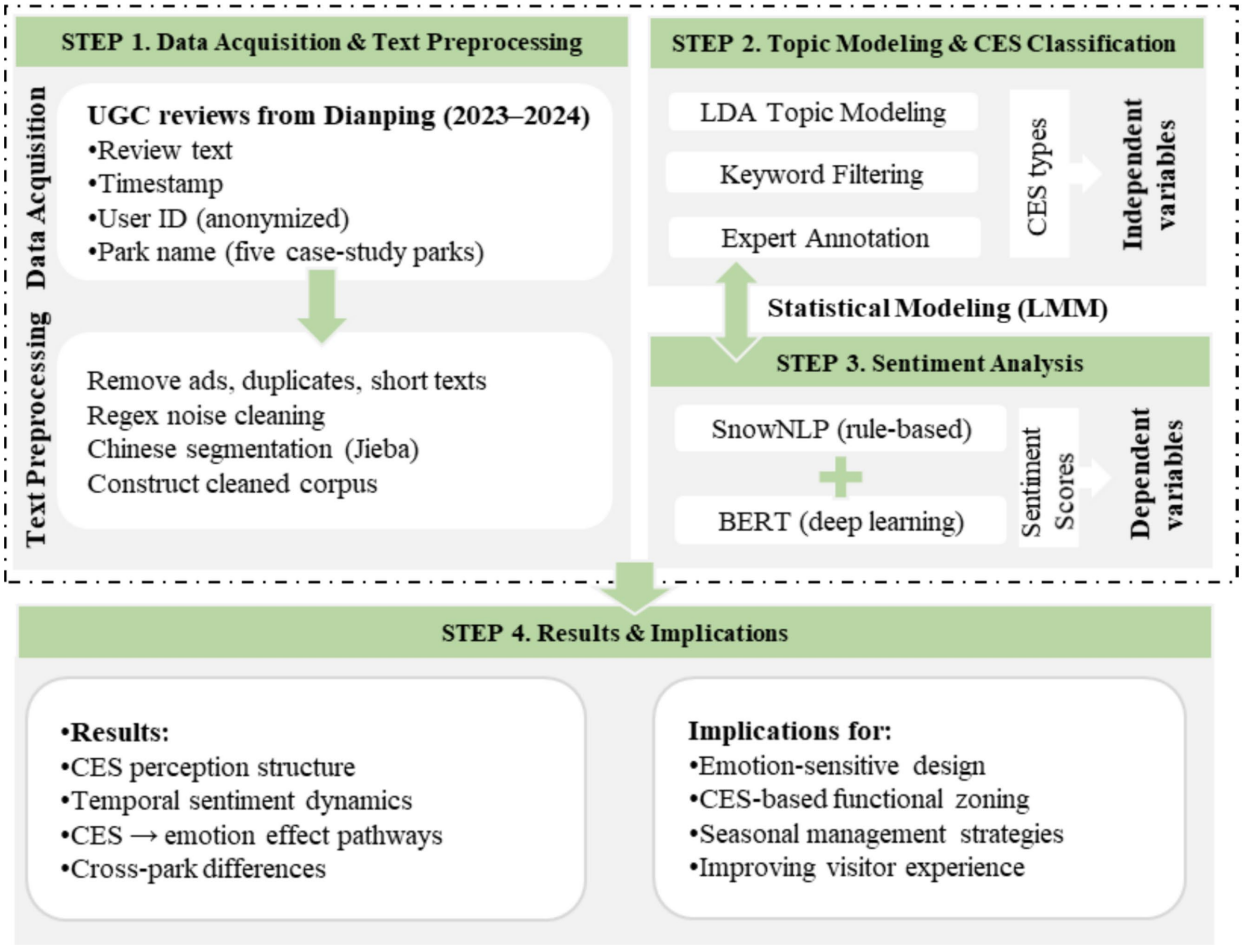
Technical roadmap of this study.

This study established an integrated analytical workflow comprising data acquisition, text preprocessing, topic identification and CES classification, sentiment analysis, and LMM-based inference (see Figure 2). User reviews of five urban parks in Shanghai were first collected from the Dianping platform and processed through cleaning, denoising, and Chinese word segmentation to construct a valid text corpus (step 1). Subsequently, an LDA model was applied to extract five latent topics, which were then aligned with five categories of CESs through expert calibration and comparison with established CES frameworks (step 2). Based on this classification, sentiment indices for each comment were computed using SnowNLP and BERT (step 3). Finally, both overall and park-specific LMMs were constructed, with CES categories treated as fixed effects and month as a random effect, to explore how different CES types influence emotional responses. This technical framework addresses the lack of CES lexicons and the ambiguity of category boundaries in Chinese-language contexts, while providing new data-driven evidence and methodological support for emotion-sensitive park planning, service optimization, and visitor experience assessment (step 4).

### 2.3 Data sources and collection methods

The data used in this study were obtained from Dianping,^1^ one of the major lifestyle-oriented social media platforms in China, which contains extensive user-generated content (UGC) related to urban parks (Liu and Xiao, 2021). To ensure a systematic and traceable data collection process, a clearly defined keyword strategy, a controlled temporal range, and standardized web-scraping procedures were adopted. In terms of the keyword search strategy, the official names of the five parks were used as the primary search terms, including “Daning Park,” “Gongqing Forest Park,” “Century Park,” “Expo Culture Park,” and “Binjiang Forest Park.” Common variants, abbreviations, and alternative expressions (e.g., “Gongqing Park,” “Century Park Scenic Area”) were added to increase coverage. To further improve recall, combined search terms such as “park name + travel note/review/comment” were incorporated. For the temporal scope, all user reviews posted between 1 January 2023 and 31 December 2024 were collected. This two-year period was selected for three reasons: (1) it represents the post-pandemic phase during which park visitation returned to high-frequency, stable levels; (2) two complete calendar years provide a sound basis for detecting seasonal patterns and interannual variations in CES perception and emotional expression; (3) review volume and UGC quality on Dianping remained relatively balanced and reliable throughout this period. Regarding web-scraping procedures, Python scripts (using requests, BeautifulSoup, and Selenium) were developed in strict compliance with research ethics and platform policies. Only publicly accessible pages were retrieved, without circumventing login requirements, captchas, or anti-scraping mechanisms. Reasonable request intervals (e.g., 3–5-s delays) and randomized user-agent strings were applied to avoid imposing unnecessary server load. Information including comment text, timestamps, anonymized user IDs, and associated park names was extracted following the page structure. All operations complied with Dianping’s Terms of Service and Robots protocol, and no non-public data or personal information was collected. The data were used exclusively for academic purposes. During the data-cleaning stage, advertisements, meaningless characters, duplicate entries, and comments fewer than five Chinese characters in length were removed to ensure data validity and analytical quality.

### 2.4 Text preprocessing and segmentation

Prior to analysis, a systematic text-cleaning and preprocessing workflow was implemented to improve the accuracy and reliability of sentiment detection and topic modeling (Siino et al., 2024). First, empty records and overly short texts (fewer than five Chinese characters) were removed to avoid misinterpretation. Next, regular expressions were applied to batch-delete noise elements such as HTML tags, URLs, emojis, user references, meaningless numbers, and system-generated characters, retaining only natural language content. Duplicate reviews were also eliminated to ensure semantic uniqueness. The cleaning process was conducted in Python, leveraging the re regular expression module, Pandas, and the Jieba segmentation tool.^2^ At the park level, the raw dataset included 2,654 reviews for Daning Park, 2,985 for Binjiang Forest Park, 7,584 for Gongqing Forest Park, 4,593 for Expo Culture Park, and 4,748 for Century Park. After cleaning and filtering, a total of 22,564 valid user reviews were retained as the data foundation for subsequent sentiment scoring and CES perception analysis (Jim et al., 2024).

### 2.5 Latent Dirichlet Allocation

Given the subjective nature of CES, users rarely employ technical terms such as “aesthetics,” “culture,” or “education” to describe their experiences. Instead, they typically express their perceptions indirectly through descriptions of leisure activities, environmental ambience, and emotional reactions (Havinga et al., 2020; Wang et al., 2021a; Xin et al., 2020). To extract latent CES-related themes from unstructured user-generated texts, this study employed Latent Dirichlet Allocation (LDA) topic modeling. LDA (Wang et al., 2018) is an unsupervised probabilistic text-mining method that treats each review as a mixture of multiple topics and represents each topic as a probability distribution over keywords, thereby revealing the underlying semantic structure of the corpus. Prior to modeling, the 22,564 valid comments were subjected to Chinese word segmentation, removal of stop words, and filtering of extremely low-frequency tokens to construct a high-quality bag-of-words model. We then conducted LDA modeling using the Gensim library in Python. Model performance under different numbers of topics was evaluated using the Coherence Score, based on which the optimal number of topics was selected. Subsequently, the high-weight keywords and semantic characteristics of each topic were compared with CES categories defined in the MEA and the CICES V5.1 framework, supplemented by relevant literature and the functional characteristics of the sampled parks (Fox et al., 2021; Huai et al., 2023; Pinto et al., 2023). Based on this comparison, manual interpretation and classification were conducted to achieve a data-driven identification of CES types.

### 2.6 Sentiment analysis

To quantify subjective sentiment tendencies expressed in user reviews (Mao et al., 2022; Wankhade et al., 2022; Yadav and Vishwakarma, 2020) and to evaluate the influence of CES on visitor sentiment, this study adopted a hybrid sentiment analysis framework that combines a rule-based model (SnowNLP) with a deep learning model (BERT). SnowNLP, based on a Naïve Bayes classifier and an internal Chinese corpus, assigns sentiment scores to review texts on a [0–1] scale, where higher scores indicate more positive sentiment. BERT (specifically, the “Erlangshen-RoBERTa-110 M-Sentiment” model released by IDEA-CCNL) leverages deep semantic feature extraction and contextual understanding to capture complex syntactic patterns and implicit sentiment. Considering the differences in algorithmic mechanisms and corpus sources, the outputs of the two models were normalized and averaged with equal weights to generate a final sentiment index. This approach balances the stability of the rule-based method with the sensitivity of the deep learning model while reducing systematic bias from relying on a single method. The final index ranges from 0 to 1 and was categorized into five levels: Strongly negative (0–0.2), Negative (0.2–0.4), Neutral (0.4–0.6), Positive (0.6–0.8), and Strongly positive (0.8–1.0). This quantitative sentiment index was then used as the dependent variable in subsequent analyses involving topic modeling, spatial features, and temporal dynamics.

### 2.7 Statistical analysis

To further examine how CES reflected in user comments influence emotional attitudes, this study employed a Linear Mixed-Effects Model (LMM) (West et al., 2022) for statistical regression analysis. Compared with ordinary linear regression, LMMs simultaneously account for fixed and random effects and are particularly suitable for data with hierarchical or nested structures—such as the month-grouped temporal structure of user comments in this study. In the model specification, the sentiment score of each comment was used as the dependent variable to represent visitors’ emotional tendencies. The CES categories identified through the LDA topic model were incorporated as binary dummy variables to serve as fixed effects (e.g., “whether the comment contains aesthetic-related content,” “whether it involves social interaction or educational services”). To control for temporal heterogeneity, month was included as a random-intercept variable, capturing potential fluctuations or shifts in emotional expression across different time periods. To more comprehensively reveal the relationship between CES and sentiment, two levels of models were constructed: (1) An overall LMM, used to evaluate the average effects of the five CES categories across all park samples; and (2) Park-specific LMMs, estimated separately for each of the five parks to identify how the emotional impacts of CES vary across different spatial contexts. This multi-level modeling design not only uncovers general mechanisms through which CES influence sentiment but also captures functional differences among parks and variations in visitors’ experiential preferences, thereby enhancing both the explanatory power and contextual relevance of the analysis (see formula below).


$${\text{Sentiment}}_{\mathrm{ij}}=\beta_{0}+\sum_{k=1}^{K}\beta_{k}\cdot {\mathrm{CES}}_{\mathrm{ijk}}+u_{j}+\in_{\mathrm{ij}}$$

Where *Sentiment_ij_* is the sentiment score of review *i* in month *j*; *CES_ijk_* indicates whether review *i* involves CES type *k* (binary variable); *β_k_* are fixed-effect coefficients; *u_j_ ∼ N(0,σ_u_^2^)* denotes the random effect of month; and *ϵ_ij_ ∼ N(0,σ^2^)* is the residual error.

In addition, to ensure that no severe multicollinearity existed among the independent variables, Variance Inflation Factor (VIF) (O’Brien, 2007) tests were conducted for the fixed effects in both the overall model and the park-specific models. The results showed that all VIF values were well below the commonly accepted threshold (< 5), indicating that multicollinearity was negligible and did not materially affect the model estimations.

## 3 Results

### 3.1 Identification of CES types

#### 3.1.1 Keyword extraction and CES classification via LDA

**Figure 3:**
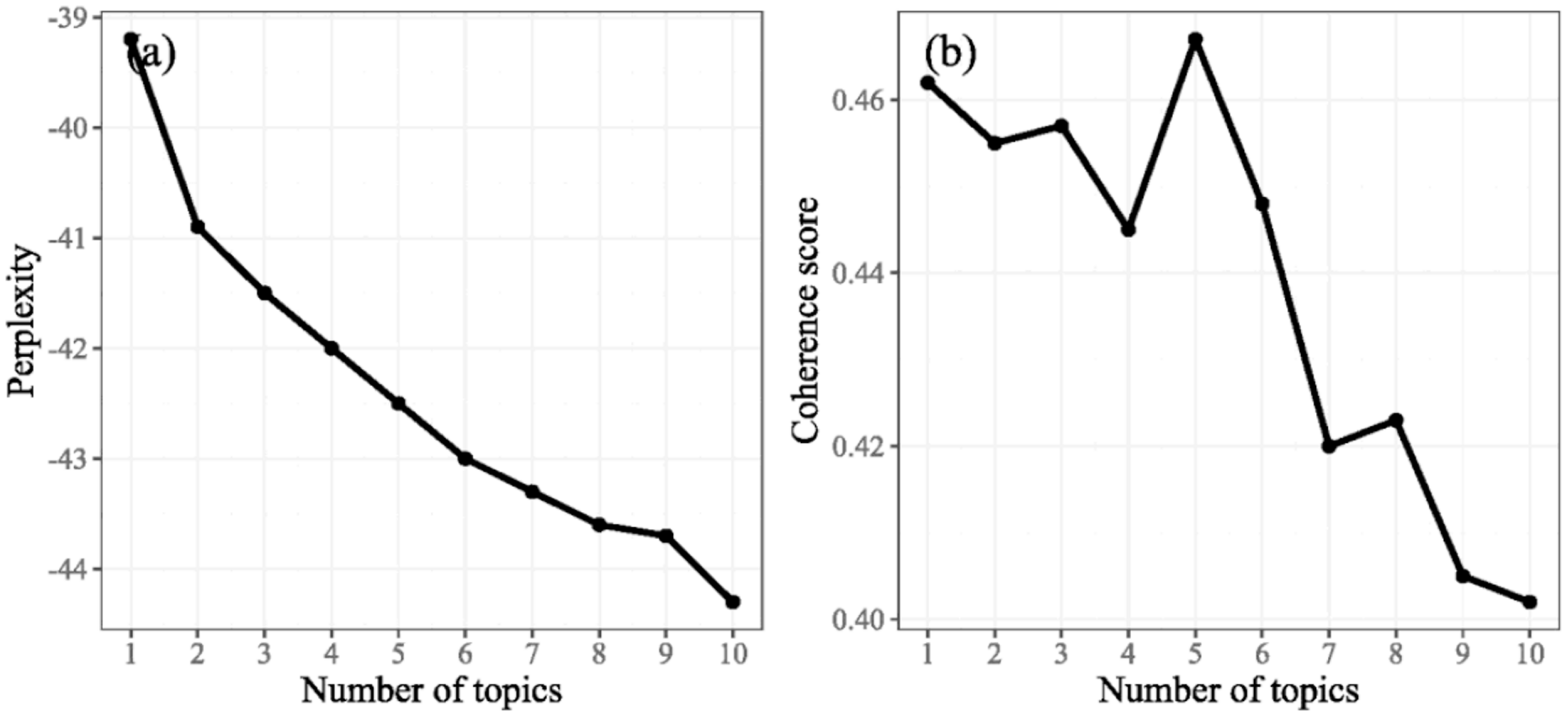
Perplexity variation **(a)** and Coherence score **(b)** variation across different topic numbers.

Building upon the frameworks of the MEA and CICES, this study applied LDA topic modeling to extract and classify the latent semantic structures of social media texts. During model training, perplexity and coherence score were calculated for different numbers of topics (*K* = 2–15) to evaluate performance in terms of model fit and semantic interpretability. Results indicated that perplexity consistently decreased with more topics, but an inflection point occurred at *K* = 5, beyond which the rate of decrease slowed, suggesting model fit stabilization. Meanwhile, coherence scores peaked at *K* = 5, reflecting the highest intra-topic consistency and semantic aggregation (Figures 3a,b). Accordingly, *K* = 5 was selected as the optimal topic number, providing a reliable foundation for subsequent identification and classification of CES types.

**Figure 4:**
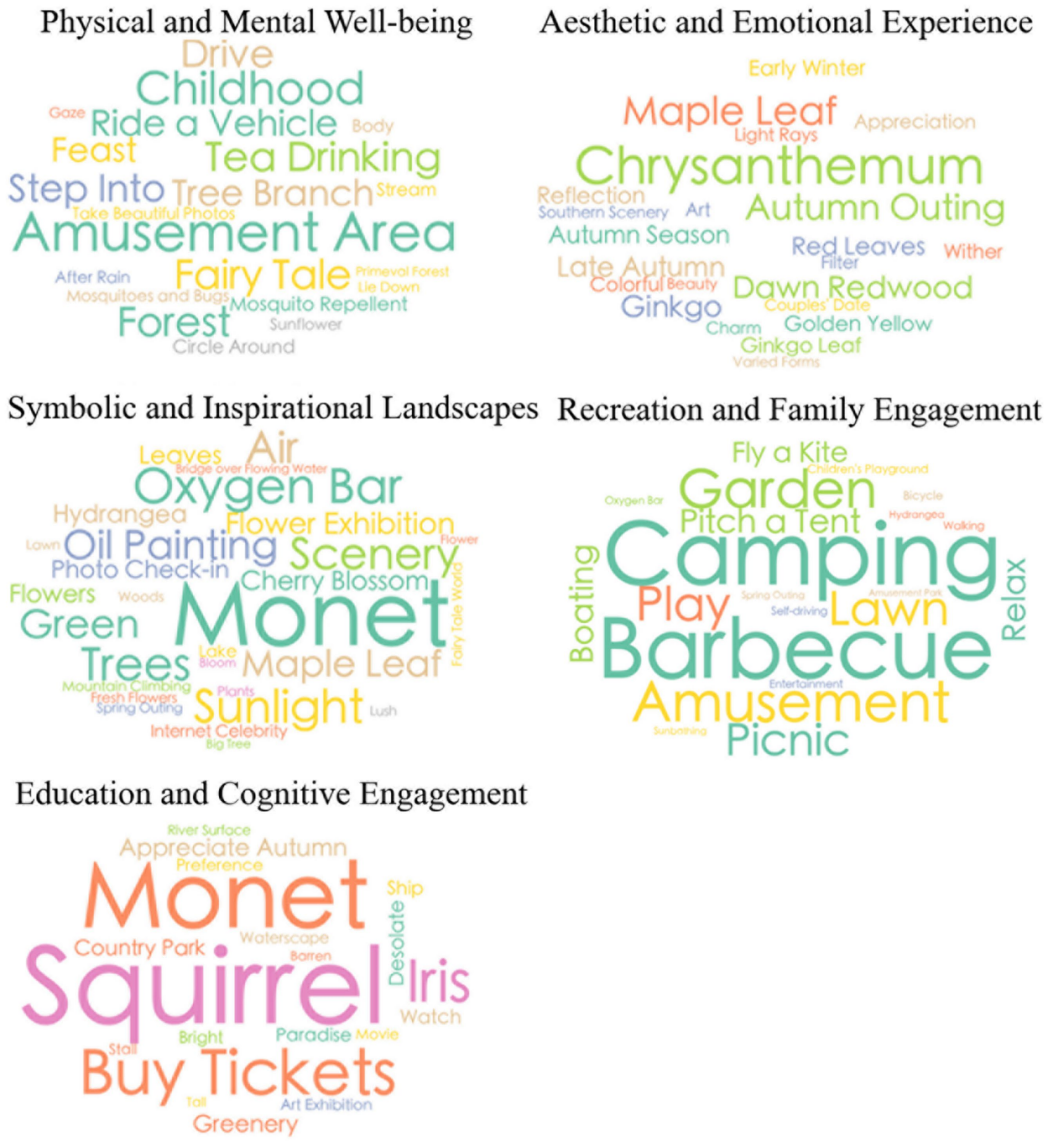
Word clouds of keywords under different CES types.

Based on the optimal number of topics (*K* = 5), the study further extracted the top 100 high-frequency keywords for each topic and conducted an in-depth analysis of their semantic characteristics. The statistical results reveal substantial thematic diversity in user comments, with high-frequency terms covering multiple core dimensions of urban park experiences—such as natural landscapes, recreational activities, seasonal features, aesthetic impressions, and education-related content. This indicates that social media text effectively captures the major functional dimensions of CES at an aggregate level. To enhance topic interpretability, the keyword statistics were examined in conjunction with the spatial layouts and common use scenarios of typical urban parks in Shanghai. Additionally, five experts with backgrounds in urban planning, ecological perception, and human geography were invited to classify and label the topics. Ultimately, the five topics extracted by the LDA model were synthesized and mapped onto five categories of CES: Physical and Recreational Health, Aesthetic and Emotional Experience, Symbolic and Inspirational Landscapes, Recreation and Family Engagement, and Education and Cognitive Engagement (see Table 1). Specifically, Physical and Recreational Health reflects public attention to sports facilities, fitness activities, and physical–mental restoration; Aesthetic and Emotional Experience captures aesthetic impressions derived from seasonal landscape changes, colors, and visual atmospheres; Symbolic and Inspirational Landscapes represents ecological features, natural elements, and immersive nature experiences; Recreation and Family Engagement describes scenarios centered on leisure, relaxation, and social interaction; and Education and Cognitive Engagement highlights the park’s role in delivering nature- and culture-related knowledge and supporting cognitive enrichment (see Figure 4).

**Table 1:** Mapping between LDA topics and CES types.

Topic no.	CES category	Functional definition	Example keywords
Topic 0	Physical and Mental Well-being	Engagement with natural environments that promote physical activity, stress relief, and mental restoration through direct contact or immersion in nature.	Walking, jogging, hiking, forest, stream, relaxation, tranquility
Topic 1	Aesthetic and Emotional Experience	Visual and emotional appreciation of nature’s beauty, color, and atmosphere, evoking aesthetic pleasure and emotional resonance.	Cherry blossom, maple, autumn, reflection, sunlight, golden leaves
Topic 2	Symbolic and Inspirational Landscapes	Nature as a symbolic, artistic, or inspirational motif reflecting cultural meaning, imagination, and creativity.	Monet, oil painting, fairytale, blooming, poetic scenery, seasonal symbolism
Topic 3	Recreation and Family Engagement	Social and leisure-oriented interactions with nature emphasizing play, relaxation, and shared experiences among family or friends.	Picnic, barbecue, cycling, playground, boating, kite flying, family outing
Topic 4	Education and Cognitive Engagement	Activities and settings that promote environmental learning, observation, and knowledge exchange about natural or cultural elements.	Exhibition, art fair, thematic show, interpretation board, nature observation, wildlife education

#### 3.1.2 Frequency distribution of CES types across parks

**Figure 5:**
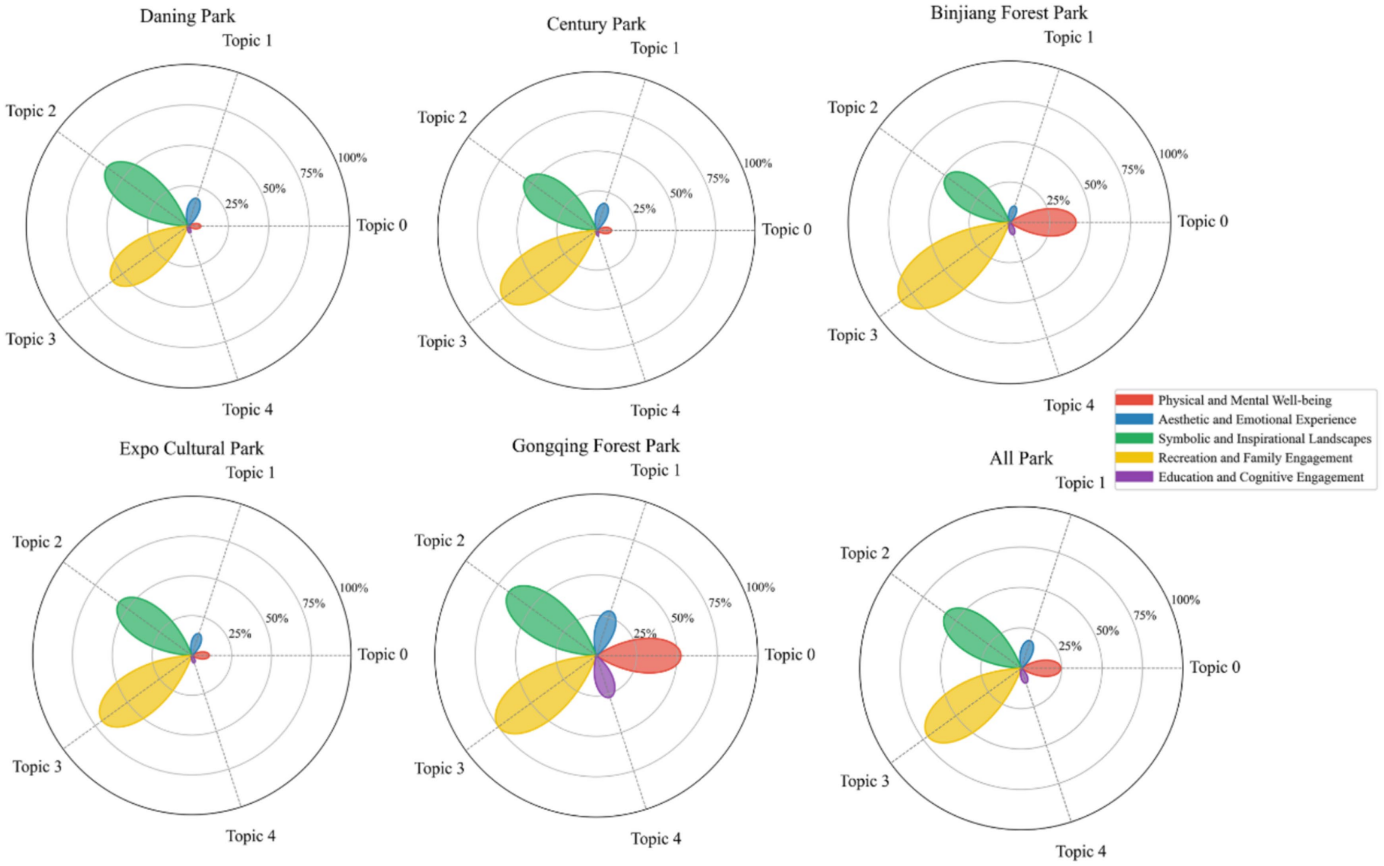
Frequency distribution of CES perceptions across the five parks.

Among all CES categories, Recreation and Family Engagement (72.70%) and Symbolic and Inspirational Landscapes (57.28%) exhibited considerably higher frequencies than Physical and Recreational Health (24.44%) and Aesthetic and Emotional Experience (17.52%), while Education and Cognitive Engagement showed the lowest frequency (9.18%) (Figure 5). For Physical and Recreational Health, Gongqing Forest Park (52.46%) and Binjiang Forest Park (41.33%) recorded relatively high frequencies, whereas the other parks were all below 11%. The frequency of Aesthetic and Emotional Experience was generally low, with Gongqing Forest Park reaching 28.83%, while the others ranged between 10.56 and 18.08%. Symbolic and Inspirational Landscapes was highly perceived across all parks, with Gongqing Forest Park (66.93%) and Daning Park (61.70%) ranking the highest, followed by Expo Cultural Park (56.47%), Century Park (54.86%), and Binjiang Forest Park (48.47%). Recreation and Family Engagement was the most frequently perceived category overall, being particularly prominent in Binjiang Forest Park (82.89%), followed by Gongqing Forest Park (74.89%), Century Park (72.50%), and Expo Cultural Park (69.67%), with Daning Park at 57.54%. By contrast, Education and Cognitive Engagement showed generally low frequencies across all parks, with Gongqing Forest Park (27.22%) displaying a noticeable presence, while the others were below 8%.

### 3.2 Distribution characteristics and comparative analysis of visitor sentiment scores

**Figure 6:**
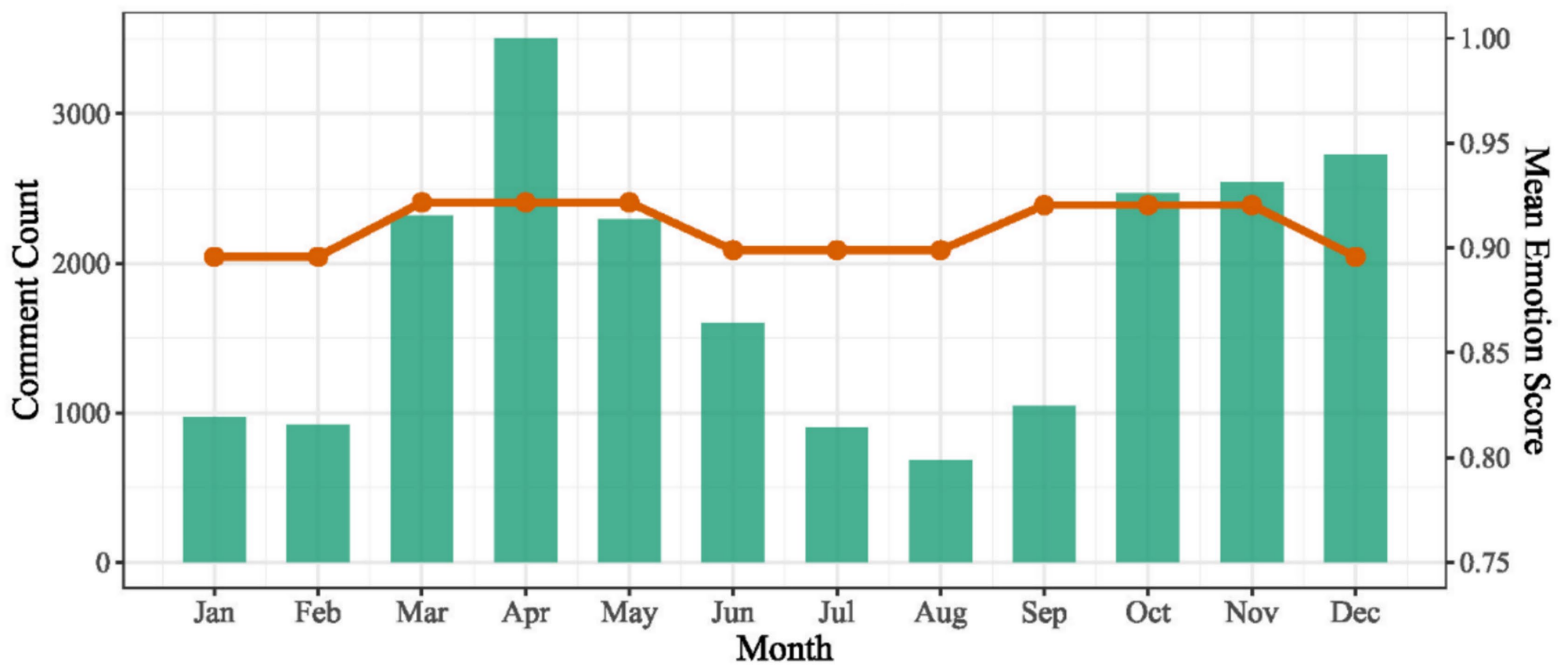
Monthly comment count and season mean emotion score.

Based on the monthly averages of sentiment scores and the distribution of comment volumes (see Figure 6), both indicators exhibit pronounced temporal fluctuations throughout the year. In terms of comment volume, user engagement is generally higher from late spring to early winter, with the lowest levels observed during summer. April (3,505 comments) and December (2,725 comments) recorded the highest monthly totals, whereas a marked decline occurred in July–August, with August showing the minimum count (682 comments). Regarding monthly sentiment scores, values remained consistently high within the range of 0.895–0.922, while still displaying noticeable month-to-month variation. Two prominent peaks emerged during the year: the first from March to May (0.922), and the second from September to November (0.921). In contrast, sentiment scores during winter (January–February and December) and summer (June–August) were comparatively lower, mostly falling within the 0.895–0.899 range. Overall, the annual sentiment trajectory exhibits a bi-modal pattern, characterized by higher emotional positivity in spring and autumn and slightly lower levels in winter and summer. Notably, changes in comment volume do not synchronize with fluctuations in sentiment, indicating that seasonal rhythms of user engagement are not directly aligned with the intensity of emotional expression.

**Figure 7:**
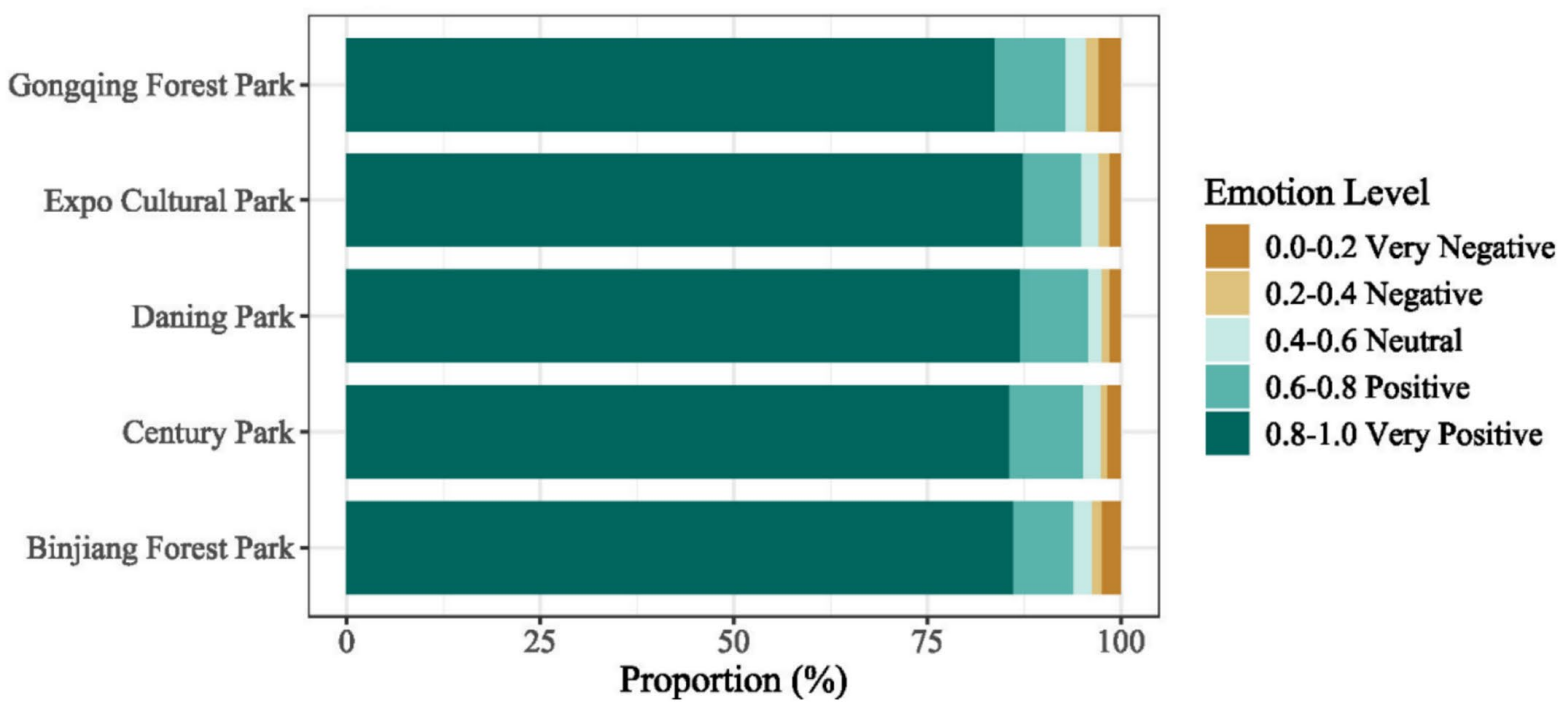
Overall distribution of visitor emotions.

Based on the classification of sentiment categories and their proportional distribution across the five urban parks (see Figure 7), notable differences can be observed among parks, although the overall emotional structure remains highly consistent. Overall, “Very Positive” (0.8–1.0) sentiment dominates across all parks, consistently exceeding 83%, indicating that visitors generally express strong positive emotions in their urban park experiences. Specifically, Expo Culture Park (87.31%) and Daning Park (86.93%) exhibit the highest proportions of “Very Positive” sentiment, followed by Century Park (85.53%) and Binjiang Forest Park (86.03%). Gongqing Forest Park shows a comparatively lower proportion at 83.60%. Meanwhile, the share of negative sentiment (“Negative” + “Very Negative”) remains low across all parks, ranging from 2.39 to 4.62%. Gongqing Forest Park records the highest proportion of negative sentiment (4.61%), whereas Daning Park (2.60%) and Century Park (2.63%) exhibit the lowest. Neutral sentiment (0.4–0.6) accounts for less than 3% of all comments in each park, suggesting that users seldom employ neutral expressions and are more inclined to convey explicitly positive attitudes in their social media narratives.

**Figure 8:**
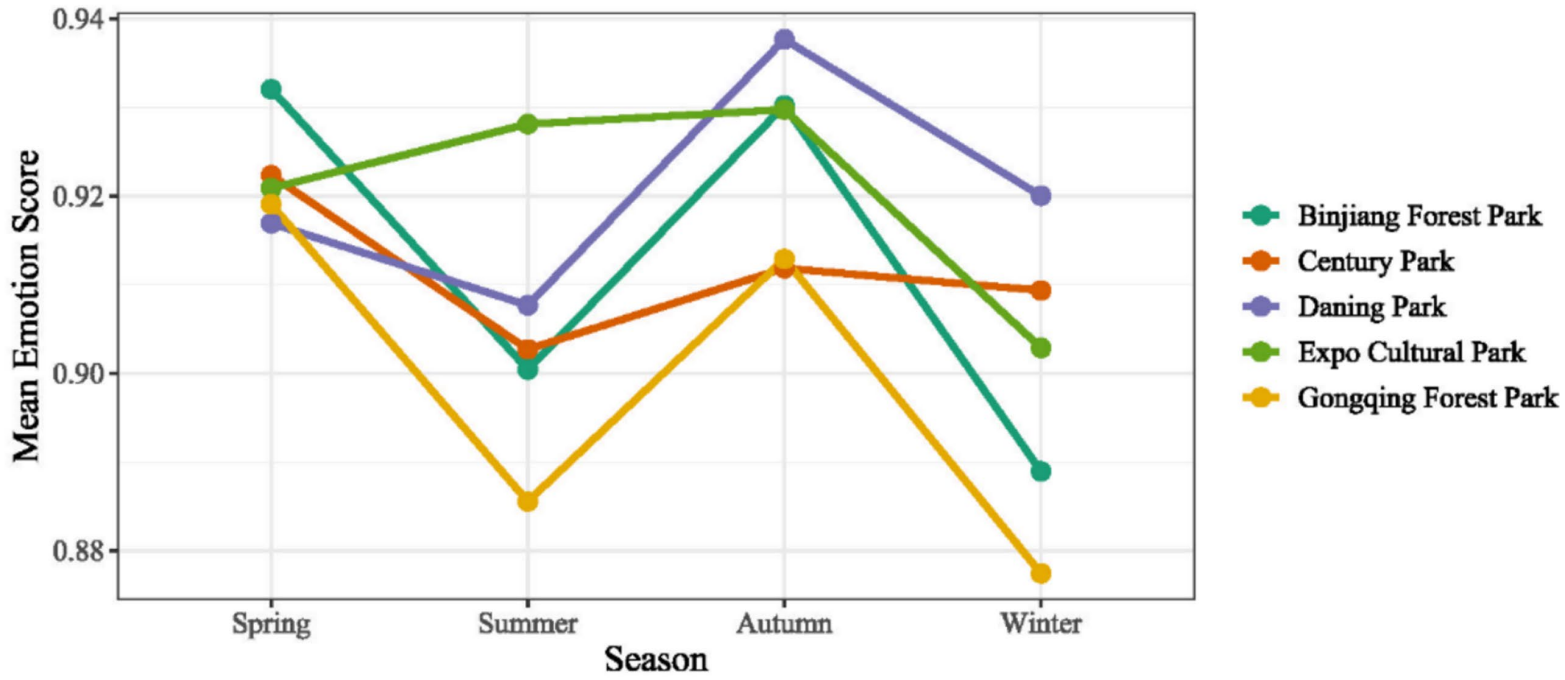
Seasonal mean emotion for each park.

Seasonal variations in sentiment (Figure 8) reveal a consistent pattern across all parks, with higher sentiment scores in spring and autumn and lower scores in summer and winter. Specifically, spring exhibits relatively elevated sentiment levels, with average scores ranging from 0.916 to 0.932 across the parks. Autumn shows a similarly strong positive emotional response, with park-level mean scores between 0.912 and 0.937, making it one of the two seasons with the highest sentiment values. In contrast, summer demonstrates generally lower sentiment levels (approximately 0.885–0.928), with Gongqing Forest Park and Binjiang Forest Park showing particularly pronounced declines. Winter displays the lowest sentiment scores among the four seasons (approximately 0.877–0.920), suggesting that colder environmental conditions and reduced vegetation coverage may weaken visitors’ emotional experiences.

### 3.3 LMMs results

Table 2 presents the effects of different CES categories on sentiment scores in both the overall model and the park-specific LMMs. Overall, Symbolic and Inspirational Landscapes and Recreation and Family Engagement consistently exhibit significant positive effects across multiple parks, indicating that content related to symbolic meaning, inspirational experiences, or leisure and family activities is more likely to evoke positive emotional responses. In contrast, Education and Cognitive Engagement shows a negative association with sentiment in the overall model and in several individual parks, suggesting that posts involving informational, analytical, or cognitively oriented content tend to yield comparatively lower sentiment scores. The park-specific models reveal differentiated patterns. For example, in Binjiang Forest Park and Gongqing Forest Park, Physical and Mental Well-being exerts a significant positive effect on sentiment, whereas in Century Park and Expo Culture Park, emotional uplift is primarily driven by symbolic, aesthetic–emotional, and family-oriented experiences. These findings highlight spatial variability in how different CES types shape emotional responses across distinct park environments.

**Table 2:** Significant effects of CES categories on sentiment scores in the overall and park-specific LMMs (only significant predictors are reported).

Park	Term	Estimate	Std.error	*p*
ALL_PARKS	(Intercept)	0.874	0.005	***
	Symbolic and Inspirational Landscapes	0.028	0.003	***
	Recreation and Family Engagement	0.040	0.002	***
	Education and Cognitive Engagement	−0.029	0.004	***
Daning Park	(Intercept)	0.905	0.006	***
	Recreation and Family Engagement	0.029	0.006	***
	Education and Cognitive Engagement	−0.041	0.018	*
Century Park	(Intercept)	0.885	0.006	***
	Symbolic and Inspirational Landscapes	0.020	0.005	***
	Recreation and Family Engagement	0.035	0.005	***
Expo Cultural Park	(Intercept)	0.892	0.005	***
	Aesthetic and Emotional Experience	0.021	0.008	**
	Symbolic and Inspirational Landscapes	0.017	0.005	***
	Recreation and Family Engagement	0.035	0.005	***
Binjiang Forest Park	(Intercept)	0.859	0.008	***
	Physical and Mental Well-being	0.020	0.007	**
	Symbolic and Inspirational Landscapes	0.027	0.007	***
	Recreation and Family Engagement	0.052	0.007	***
Gongqing Forest Park	(Intercept)	0.829	0.007	***
	Physical and Mental Well-being	0.025	0.005	***
	Symbolic and Inspirational Landscapes	0.052	0.005	***
	Recreation and Family Engagement	0.053	0.005	***
	Education and Cognitive Engagement	−0.033	0.006	***

The significance symbols represent the following levels: *p < 0.05, **p < 0.01, ***p < 0.001.

Table 3 reports the standard deviations of the random effects in both the overall model and the park-specific models, including the month-level random intercepts (Month-level SD) and the observation-level residuals (Residual SD). The results show that the standard deviations of the monthly random intercepts are relatively small (0.010–0.020), indicating that month, as a random factor, contributes only minimally to the variability in sentiment scores. In other words, differences in average sentiment across months are relatively stable and limited. In contrast, the observation-level residual standard deviations (0.157–0.193) are substantially larger than the month-level variances. This suggests that most of the variation in sentiment scores originates from individual comments themselves, rather than from macro-level temporal factors such as month. Notably, Gongqing Forest Park and Binjiang Forest Park exhibit slightly higher month-level and residual variability compared with the other parks, indicating greater heterogeneity in emotional expression among users in these areas. In contrast, Expo Culture Park shows the smallest month-level fluctuation (SD = 0.010), suggesting that sentiment in this park is the most stable across months.

**Table 3:** Standard deviations of random effects in the overall and park-specific LMMs.

Random effect	ALL_PARKS	Daning Park	Century Park	Expo Cultural Park	Binjiang Forest Park	Gongqing Forest Park
Month-level SD (intercept)	0.015	0.012	0.015	0.010	0.017	0.020
Residual SD (observation)	0.176	0.157	0.164	0.161	0.181	0.193

## 4 Discussion

### 4.1 Public perceptions of CES preferences and semantic features

This study reveals a pronounced imbalance in the distribution of CES perceptions across Shanghai’s urban parks. Overall, Physical and Recreational Health and Symbolic and Inspirational Landscapes are the two most frequently perceived CES categories, whereas Education and Cognitive Engagement appears least prominent. In large multifunctional parks such as Binjiang Forest Park and Gongqing Forest Park, themes related to physical well-being, natural scenery, and symbolic or inspirational experiences dominate user comments and are associated with higher sentiment scores. This pattern aligns with existing research showing that natural elements—such as forests and water bodies—provide multiple benefits, including aesthetic enjoyment, stress reduction, and enhanced well-being (Phillips et al., 2022). In contrast, although Physical and Recreational Health and Aesthetic and Emotional Experience remain important for specific user groups, their overall comment frequency is comparatively lower, possibly due to their more specialized usage scenarios and narrower user base.

A particularly noteworthy finding is the consistently low frequency of Education and Cognitive Engagement across most parks. On the one hand, educational content is commonly delivered through interpretive panels, science outreach events, or informational displays, which tend to be less interactive and less emotionally stimulating than natural scenery or recreational activities. On the other hand, social media posts typically emphasize immediate experiential impressions rather than deeper cognitive processes or learning outcomes, limiting the visibility of education-related CES in text-based data. To enhance the reliability of CES identification, this study did not rely solely on the raw outputs of the LDA model. Instead, a three-step procedure—keyword filtering, semantic validation, and expert calibration—was adopted. First, the top 100 high-probability keywords from each topic were extracted, followed by the removal of function words, noise words, and terms with weak relevance to park experiences. Second, manual contextual checks were performed by cross-referencing the spatial and functional characteristics of the selected parks to ensure that each keyword aligned semantically with its assigned CES type. Finally, five experts with backgrounds in urban planning, ecological perception, and human geography independently annotated the keywords. Terms with multiple potential meanings were discussed collectively, and their classification was determined based on original textual context and dominant semantic orientation. This process effectively strengthened the robustness of the keyword–CES mapping and reduced biases arising from semantic ambiguity.

Moreover, to improve the interpretability of CES classification, particular attention was given to activity-related keywords that could potentially span multiple CES categories. For instance, terms such as “cycling” and “boating” may involve physical exertion, yet semantic co-occurrence analysis showed that in our corpus they appeared predominantly in leisure-oriented, family-based, or social interaction contexts, often alongside expressions such as “family outing,” “weekend trip,” or “playing with children.” In contrast, their co-occurrence with fitness- or training-related expressions was minimal. Therefore, these terms were categorized under Recreation and Family Engagement rather than Physical and Mental Well-being, reflecting their dominant experiential meanings within the social media text. This contextualized classification approach helped reduce ambiguity and ensured a closer alignment between CES categories and real-world user experiences.

Overall, the findings indicate that visitors’ preferences in Shanghai’s urban parks cluster strongly around Aesthetic and Emotional Experience and Symbolic and Inspirational Landscapes, two CES categories closely associated with scenic beauty and immersive nature experiences. This pattern may reflect broader influences such as residents’ lifestyles, aesthetic preferences, and the functional positioning of urban parks (Li et al., 2023; Wu et al., 2023). However, certain CES types—such as educational or cultural-identity-related services—remain inherently less visible in social media texts due to subtle expression, low textual frequency, and limited interaction within the park experience itself. These limitations underscore the need for future research to incorporate multi-source and multi-method approaches—including interviews, surveys, participatory mapping, and field observations—to complement text mining and more comprehensively capture the multidimensional structure of CES perceptions.

### 4.2 General features of sentiment responses and spatiotemporal mechanisms

Visitor sentiment scores in Shanghai’s urban parks were predominantly positive, with “positive” and “strongly positive” reviews consistently comprising the majority across all months. This pattern aligns with previous studies (Addas and Maghrabi, 2022; Cheng et al., 2021), and underscores the essential role of urban green spaces in psychological restoration and the elicitation of positive emotional responses. Instances of strongly negative sentiment were rare, reflecting the overall stability and quality of park services and indicating the absence of systemic problems or widespread negative visitor experiences (Li et al., 2025). Although differences among parks were relatively modest, notable variations were still evident. Century Park and Daning Park exhibited the highest and most concentrated sentiment scores, suggesting comparative advantages in landscape design, facility provision, and the consistency of visitor experiences. In contrast, Binjiang Forest Park and Expo Culture Park showed slightly lower means and greater variability, likely reflecting their distinct functional roles. For example, Binjiang Forest Park benefits from its waterfront landscape but faces constraints related to accessibility and service facilities; Expo Culture Park, as a newly developed green space, is still in the process of establishing its multifunctional features and spatial vitality (Li et al., 2024). Thus, variation in sentiment scores captures not only the overall emotional level but also differences in the stability of user experiences shaped by park-specific spatial and functional attributes.

Temporally, sentiment scores remained consistently positive throughout the year. Nonetheless, slight increases in “neutral” and “negative” reviews during certain months suggest that visitor perceptions may be influenced by environmental or seasonal conditions. Periods characterized by prolonged rainfall, extreme heat, or reduced ecological quality—such as during off-peak tourism seasons—may diminish the favorability of park experiences and trigger more negative feedback. This observation aligns with previous findings that the restorative effects of green spaces are sensitive to seasonal climatic conditions and ecological states (Liang and Zhang, 2021; Linwei et al., 2021; Zhang et al., 2022).

### 4.3 Mechanisms of CES influence on visitor sentiments

Compared with the growing body of studies that use social media data to analyze CES and visitor emotions in urban parks (Huai et al., 2022; Huai and Van de Voorde, 2022; Wang et al., 2016), this study offers three key innovations.

First, the study employs a structured and theory-informed approach to topic interpretation. Unlike conventional methods that rely primarily on subjective interpretation of LDA-derived keywords, we integrate high-frequency keyword statistics, spatial-functional characteristics of the sampled parks, and typical use scenarios. Furthermore, five experts in urban planning, ecological perception, and human geography participated in the topic labeling and review process. The resulting five CES categories—Physical and Recreational Health, Aesthetic Experience, Natural Landscape, Leisure and Entertainment, and Environmental Education—exhibit strong theoretical coherence and contextual relevance, providing a more systematic representation of cultural experiences in urban parks. This “topic modeling + expert semantic validation” framework enhances the stability, interpretability, and cross-spatial applicability of topic classification, addressing limitations in existing studies that depend heavily on subjective judgment (Gaikwad and Shinde, 2019; Huai and Van de Voorde, 2022).

Second, this study constructs a quantitative relationship between CES and emotional responses through LMMs. Unlike traditional regression or descriptive sentiment methods, LMMs account for temporal heterogeneity by incorporating month as a random effect, thereby reducing seasonal bias and improving model suitability for long-term, multi-park social media data (Daniel et al., 2012; Hernández-Morcillo et al., 2013). The overall model reveals that CES categories such as Symbolic and Inspirational Landscapes, Recreation and Family Engagement, and Education and Cognitive Engagement exert significant influences on sentiment. Park-specific models further uncover distinct patterns across different spatial contexts: for example, Recreation and Family Engagement consistently produces positive effects in most parks, whereas Symbolic and Inspirational Landscapes shows particularly strong effects in parks with pronounced natural or ecological features. Notably, Education and Cognitive Engagement demonstrates a significant negative association with sentiment in the overall model and several individual parks. This may be attributed to the informational nature of educational features—such as exhibits, interpretive signage, and thematic activities—where visitors tend to provide more neutral, evaluative, or mildly critical comments on content quality, presentation, or maintenance. Moreover, educational experiences are typically not the primary motivation for park visits and evoke weaker emotional responses than scenery appreciation, recreation, or family interaction, making them more prone to comments such as “slightly disappointing,” “average,” or “not as expected.” Taken together, the LMM framework enhances the statistical rigor of CES–emotion inference and provides a scalable approach for cross-site comparisons.

Finally, by applying an integrated LDA–LMM analytical framework to typical parks in Shanghai, this study contributes new empirical evidence for understanding cultural needs and emotional feedback related to nature use in megacities. The findings highlight differentiated emotional effects of CES types in everyday recreational contexts, advancing the shift from merely describing emotions to uncovering underlying drivers of emotional responses. These insights offer quantifiable references for urban green space design, public service provision, and landscape experience optimization.

### 4.4 Implications for urban park management

By integrating LDA topic modeling with expert semantic annotation, this study systematically mapped social media text content onto five categories of CES. Among them, Recreation and Family Engagement and Symbolic and Inspirational Landscapes exhibit the most significant positive effects on sentiment in the overall LMM, and these effects remain consistent across park-specific sub-models. This finding suggests that interactive, participatory, and immersive landscapes and activity spaces are central to enhancing positive emotional experiences in urban parks (Gottwald et al., 2022; Huai and Van de Voorde, 2022). Accordingly, future park planning should consider CES as a direct basis for functional zoning, prioritizing improvements to leisure and social spaces, culturally expressive landscapes, and immersive ecological environments to promote more emotionally supportive park design.

The study also identified a distinct bi-modal seasonal pattern in emotional responses: sentiment scores peak in spring and autumn and decline in summer and winter. Notably, temporal variations in sentiment do not align with fluctuations in comment volume, indicating that changes in visitor numbers alone cannot explain differences in emotional expression. This suggests the need for season-sensitive management strategies. For example, during high-sentiment seasons (spring and autumn), park managers could enhance seasonal vegetation displays or organize thematic events. Conversely, in low-sentiment seasons (summer and winter), improvements such as enhanced shading, extended evening hours, winter lighting, or evergreen planting can help mitigate seasonal declines in emotional well-being (Cheng et al., 2022; Márquez et al., 2023; Wu et al., 2023). Analysis of emotion categories further reveals that although overall positive sentiment exceeds 83% in all parks, inter-park differences remain. Gongqing Forest Park shows the highest proportion of negative sentiment, whereas Expo Culture Park and Daning Park display the highest share of “Very Positive” responses. CES-specific patterns also vary: natural landscape experiences dominate in forest parks, while recreational and entertainment experiences are more prominent in waterfront or open-structured parks. These findings highlight the importance of avoiding one-size-fits-all management and instead adopting differentiated strategies tailored to each park’s functional orientation, ecological features, and user characteristics—for instance, enhancing ecological trails and safety in natural parks, or improving activity capacity and public facilities in comprehensive parks. Finally, the LMM results quantify the emotional effects of different CES types, providing a methodological foundation for developing data-driven monitoring systems for urban parks. In the future, real-time emotion monitoring based on social media text could be used to assess the immediate emotional impacts of facility upgrades, event organization, or seasonal environmental changes. Moreover, developing CES–emotion coupling indices may help evaluate the alignment between cultural service provision and emotional feedback, supporting annual performance assessments. Such data governance frameworks would facilitate a transition from traditional experience-based management to emotion-informed, precision-based urban park governance.

### 4.5 Limitations and future directions

This study has several limitations. First, although this research proposes a CES classification framework suitable for the Chinese context by combining LDA modeling, keyword statistics, and expert semantic annotation, social media text cannot fully replace traditional surveys or experimental data. Expert annotations and text modeling inevitably involve subjectivity and uncertainty. Therefore, future studies should validate the robustness and generalizability of this framework using datasets from additional regions. Second, the dataset relies on user-generated content from platforms such as Dianping, whose users tend to be younger and more highly educated urban residents. This may introduce demographic bias and limit the representativeness of broader social groups. Furthermore, the short, colloquial, and sometimes ambiguous nature of social media text may lead to underestimation of certain CES types—such as inspirational, educational, or place-based services. Sentiment models may also struggle with implicit or multi-layered emotional expressions. Third, the LDA model has structural limitations. Some high-frequency keywords carry multiple semantic orientations—for example, “taking photos” may reflect both leisure activities and aesthetic appreciation, while “cherry blossoms” refer simultaneously to natural landscapes and aesthetic imagery. Although expert annotation helped determine primary semantic alignment, partial overlap between CES categories remains inevitable and may affect the precision of statistical estimates. In addition, some activity-related keywords inherently carry multiple semantic orientations. Although contextual analysis was used to determine dominant meanings, a degree of ambiguity may remain, which could affect the precision of CES classification. Future research could incorporate more advanced semantic embedding models or multi-label classification frameworks to enhance semantic stability. Finally, the LMM includes only month as a random effect to account for temporal heterogeneity. Additional factors—such as local environmental quality, vegetation cover, holidays, or visitor volume—were not incorporated, which limits deeper interpretation of the mechanisms underlying emotional dynamics. Future work should integrate multi-source data (e.g., street-view imagery, environmental monitoring, mobility data) and construct higher-dimensional multi-level models to provide a more comprehensive representation of emotional experiences.

## 5 Conclusion

Based on large-scale social media text from five representative urban parks in Shanghai, this study developed an integrated framework combining LDA topic modeling, a CES classification system, sentiment analysis, and LMM inference to quantify visitors’ perceptions of CES, assess their emotional impacts, and reveal temporal patterns and inter-park differences. First, CES perceptions exhibit marked imbalance. The LDA model identified five CES categories, among which Recreation and Family Engagement and Symbolic and Inspirational Landscapes appeared most frequently, reflecting the dominant roles of leisure–social activities and nature-based experiences in urban parks. In contrast, Education and Cognitive Engagement showed the lowest frequency and increased only slightly in parks with explicit science popularization features, indicating its limited visibility and relatively weak expression in user-generated text. Second, visitor sentiment was consistently positive but varied across seasons and parks. Sentiment scores remained high throughout the year (0.895–0.922), with significantly higher values in spring and autumn, forming a clear “dual peak” pattern. Across all parks, the proportion of Very Positive sentiment exceeded 83%, and negative sentiment was minimal, demonstrating the sustained mood-enhancing function of urban parks. Although minor inter-park differences were observed, overall emotional structures remained highly stable, largely driven by seasonal landscape characteristics and activity rhythms. Third, CES types exhibited distinct emotional effects. LMM results show that Symbolic and Inspirational Landscapes and Recreation and Family Engagement significantly enhanced sentiment in the overall model and in most park-specific models. Physical and Mental Well-being also showed positive effects in forest-type parks. In contrast, educational CES showed slight negative effects in the overall model and several parks, likely because text related to educational content tends to be more evaluative or descriptive rather than emotionally expressive. In summary, this study not only uncovers key patterns in CES perceptions and emotional responses in Shanghai’s urban parks but also clarifies the differentiated emotional mechanisms associated with different CES types. The proposed analytical framework effectively addresses challenges related to ambiguous CES classification and implicit textual expression in the Chinese context, providing quantitative evidence and practical insights for enhancing park experiences, optimizing functional configurations, and promoting emotionally supportive urban park planning.

## Data Availability

The original contributions presented in the study are included in the article/supplementary material, further inquiries can be directed to the corresponding author.

## Footnotes

1
https://www.dianping.com/

2
https://github.com/fxsjy/jieba
